# Warmer temperatures limit the effects of antidepressant pollution on life-history traits

**DOI:** 10.1098/rspb.2021.2701

**Published:** 2022-02-09

**Authors:** Lucinda C. Aulsebrook, Bob B. M. Wong, Matthew D. Hall

**Affiliations:** School of Biological Sciences, Monash University, Melbourne, VIC 3800, Australia

**Keywords:** pharmaceutical pollution, thermal stress, multi-stressor, fluoxetine, life history, *Daphnia magna*

## Abstract

Pharmaceutical pollutants pose a threat to aquatic ecosystems worldwide. Yet, few studies have considered the interaction between pharmaceuticals and other chronic stressors contemporaneously, even though the environmental challenges confronting animals in the wild seldom, if ever, occur in isolation. Thermal stress is one such environmental challenge that may modify the threat of pharmaceutical pollutants. Accordingly, we investigated how fluoxetine (Prozac), a common psychotherapeutic and widespread pollutant, interacts with temperature to affect life-history traits in the water flea, *Daphnia magna*. We chronically exposed two genotypes of *Daphnia* to two ecological relevant concentrations of fluoxetine (30 ng l^−1^ and 300 ng l^−1^) and a concentration representing levels used in acute toxicity tests (3000 ng l^−1^) and quantified the change in phenotypic trajectories at two temperatures (20°C and 25°C). Across multiple life-history traits, we found that fluoxetine exposure impacted the fecundity, body size and intrinsic growth rate of *Daphnia* in a non-monotonic manner at 20°C, and often in genotypic-specific ways. At 25°C, however, the life-history phenotypes of individuals converged under the widely varying levels of fluoxetine, irrespective of genotype. Our study underscores the importance of considering the complexity of interactions that can occur in the wild when assessing the effects of chemical pollutants on life-history traits.

## 1. Introduction

Pharmaceutical pollution has become an increasing threat to ecosystems worldwide. Thousands of pharmaceuticals are used for medical and veterinary healthcare, and more than 600 have been detected in the environment [1]. A large number of these remain bioactive when excreted [2], and sewage treatment is often insufficient at removing these products [3]. Once in the environment, pharmaceuticals are often resistant to degradation [2] and have the ability to bioaccumulate and transfer through food webs [4]. As many pharmaceuticals are designed to elicit responses at low doses [5], and often target receptors that are evolutionarily conserved in a range of species [6], there are mounting concerns about how these drugs may be affecting populations in the wild [7,8].

One such pharmaceutical of concern is fluoxetine, the active ingredient of Prozac—one of the most commonly prescribed antidepressants in the world [9]. Fluoxetine has been found in aquatic systems globally [4,9,10], with levels as high as 500 ng l^−1^ downstream from wastewater treatment plants [11]. Fluoxetine functions as an antidepressant by increasing the effect of the neurotransmitter serotonin by inhibiting transport proteins that reuptake serotonin [12]. Antidepressants that function in such a manner are classed as selective serotonin reuptake inhibitors (SSRIs) [12,13]. The target molecule of fluoxetine (serotonin transporter, 5-HTT) is present in a wide variety of taxa [14], which gives fluoxetine the potential to affect non-target organisms. In fact, studies have shown that fluoxetine exposure can cause adverse effects in a range of aquatic organisms, including reduced fecundity in snails [15], impaired development in tadpoles [16] and disturbed behaviour in fish [17–19]. Few studies, however, have examined the impacts on life-history traits at field-relevant concentrations, which is surprising considering that changes in these traits are expected to have dire fitness and evolutionary consequences [7,20,21].

While many studies have shown that pharmaceuticals can affect wildlife, these typically investigate the direct effects of single toxicants on organisms and do not consider how pharmaceutical pollutants can interact with other stressors [22]. This limits our understanding of the ecological impacts of these pollutants, as in natural environments, organisms are exposed to a variety of biotic and abiotic stressors simultaneously, and the combined effects of stressors are rarely additive [23,24]. Instead, stressors commonly interact to produce effects markedly different from the sum of each isolated effect, which can cause the true ecological impact of stressors to be misinterpreted [25,26]. For example, synergistic interactions between chemical pollutants and secondary stressors are frequently reported [27–30], perhaps due to the energetic cost of detoxification resulting in wildlife more vulnerable to secondary stressors. Conversely, antagonistic interactions could lessen the damage caused by pharmaceuticals, due to one stressor enabling tolerance to another, or having opposing effects [31]. The severity of the impact of a pharmaceutical pollutant on an ecosystem may, therefore, depend on what other stressors are present in the environment, a notion overlooked by the majority of studies.

One important stressor to consider is temperature, due to its direct effects on growth, reproduction and development of organisms, particularly ectotherms [32]. Furthermore, global change is causing temperature to become an increasingly concerning stressor to many ecosystems [33]. Increased temperature is often shown to exacerbate the effects of pollutants [28,34,35], via increasing toxicity [36] or reproductive costs [37]. While less common, there are also cases of heat stress reducing the effects of pollutants [28,38,39], through temperature and pollutants having opposing effects [40], or temperature disrupting the mechanism responsible for the pollutant's effects [41]. Nevertheless, few studies have investigated interactions between increased temperature and exposure to psychoactive pharmaceutical pollutants such as fluoxetine.

Another limitation of the majority of studies on pharmaceutical pollution is that they typically examine the effects on individual traits only [5,15]. While these studies provide important insights into how organisms may be impacted by pollutants, they can lead to contrasting or confusing results because the changes in phenotype may vary in magnitude or direction depending on the specific trait being measured [42]. In reality, organism phenotypes are comprised numerous traits, many covarying and capturing the full extent of environmental interactions requires understanding shifts in these integrated phenotypes [43,44]. Phenotypic trajectory analysis can achieve this through exploring phenotype shifts across multivariate space, allowing a more holistic understanding of how stressors may affect a population [45,46].

Here, we examined the consequences of fluoxetine exposure on life-history trait phenotypes under differing temperature treatments using *Daphnia magna*, a commonly used model organism in ecotoxicology [47]. The temperature treatments used were 20°C, which is the standard cultivation temperature of *Daphnia* [26,48–50], and 25°C, which is known to affect ‘pace-of-life’ traits, often leading to significantly faster maturation, earlier offspring release and smaller size at maturity, but at the expense of reduced survival and lifetime fecundity (e.g. [51–53]). For our fluoxetine exposure treatments, we compared a freshwater control with two environmentally realistic concentrations: 30 ng l^−1^, representing levels commonly detected at surface waters and 300 ng l^−1^, which represents approximate concentrations detected at wastewater outlets [54]. In addition, we included an extreme concentration of 3000 ng l^−1^ as a comparison to levels commonly used in acute toxicity tests (e.g. [55–57]), as well as a freshwater control with no fluoxetine. Using a variety of life-history traits, we then employed phenotypic trajectory analysis to compare the magnitude and direction of temperature-induced phenotypic shifts for *Daphnia* under varying fluoxetine exposures, allowing us to assess whether increased temperature might intensify or reduce the effects of pharmaceutical pollutants on wildlife.

## 2. Methods

### (a) Study system

*Daphnia magna* is a freshwater filter-feeding crustacean native to Eurasia. The species is a model organism in both evolutionary biology and aquatic toxicology as it reproduces rapidly, is sensitive to their chemical environment and plays an essential role in freshwater ecosystems as primary consumers [48]. *Daphnia magna* most frequently produce asexually via cyclic parthenogenesis [48], resulting in genetic clones, which allow stocks of single genotypes to be easily maintained in a laboratory environment. For the current study, we used two *Daphnia* genotypes derived from single clones: HU-HO-2 (herein HO2) from Hungary and BE-OHZ-M10 (herein M10) from Belgium. These geographically diverse clones are known to vary in several life-history traits (e.g. [49,50,58]), and allow us to investigate whether fluoxetine and temperature effects are likely to be genotype specific.

Prior to the experiment, three generations of *Daphnia* were housed individually in 70-ml jars filled with 45 ml of artificial *Daphnia* media [59,60]. The medium was replaced twice a week and each jar was fed daily with an ad libitum amount of algae (*Scenedesmus* spp.). Food levels were gradually increased in accordance to the needs of the animals, from 0.5 million cells per animal on day 1, to 5 million cells per animal from day 8 onwards. All animals were kept in incubators with an 18 : 6 h light–dark cycle at a fixed temperature of 20°C. Experimental animals were taken from clutch 3–4 of 126 parental *Daphnia* of each genotype. These were maintained under the same standard conditions as parental lines, with the exception of temperature, which was fixed at either 20°C or 25°C depending on treatment group.

### (b) Fluoxetine and temperature exposure

We used a factorial experimental design where the two *Daphnia* genotypes (M10 and HO2) were exposed to the two temperature treatments (20°C or 25°C), under the four different nominal fluoxetine concentrations (0 ng l^−1^, 30 ng l^−1^, 300 ng l^−1^ and 3000 ng l^−1^). Twenty individuals were used for each genotype–temperature–fluoxetine treatment combination. Fluoxetine treatments were produced by dissolving the desired amount of fluoxetine hydrochloride in small volume of methanol, as per previously established protocols [18,61,62], then dosing this methanol into media before distributing the media across jars. The media for the control treatment was dosed with a similar volume of methanol, but with no fluoxetine hydrochloride. All animals were exposed to fluoxetine treatments at 1 day old, and fluoxetine dosing occurred at each water change (i.e. twice weekly).

At each water change, samples were drawn from fluoxetine-dosed media to monitor fluoxetine concentrations, with the measured effective concentrations after the exposure period in line with the initial nominal fluoxetine concentrations doses (25.87 ± 2.47 ng l^−1^, 197.5 ± 10.31 ng l^−1^ and 1900 ± 184.39 ng l^−1^, see electronic supplementary material). Water analysis was performed by Envirolab Services (MPL Laboratories; NATA accreditation: 2901; accredited for compliance with ISO/IEC: 17025) using gas chromatography-tandem mass spectrometry (7000C Triple Quadrupole GC-MS/MS, Agilent Technologies, Delaware, USA) following methods described in [62].

Individuals were monitored daily for survival and the number of offspring and clutches produced was counted twice a week at each water change. Fecundity was calculated as the total number of offspring produced by each individual during the course of the experiment. The experiment was terminated at 30 days, whereupon the body size of all remaining *Daphnia* was measured as the length of *Daphnia* from the top of the head above the eye to the base of the tail spine. Intrinsic rates of increase per individual (*r*) were calculated using the timing and number of offspring and then solving the Euler–Lotka equation (following [63]).

### (c) Statistical analysis

Statistical tests were conducted using R software v. 4.0.3 software [64]. We first implemented linear mixed-effect models for each of the life-history traits measured, using fluoxetine treatment, temperature treatment, genotype and interactive terms as fixed effect factors, and blocks as a random effect. Across all traits, we then performed a phenotypic trajectory analysis (PTA) in order to determine how temperate and fluoxetine interact to shape a life-history phenotype [44]. This approach quantifies the relative magnitude (*D*) and angle (*θ*) of any shift in multivariate phenotype (phenotypic trajectory) across temperature for each fluoxetine concentrations using a permutation-based MANOVA. Multivariate analyses were conducted using the RRPP package [65], traits were scaled to a mean of 0 and standard deviation of 1, and subsequently visualized using principal component analysis (PCA).

## 3. Results

### (a) Effects of fluoxetine vary by trait, genotype and temperature

**Figure 1.**
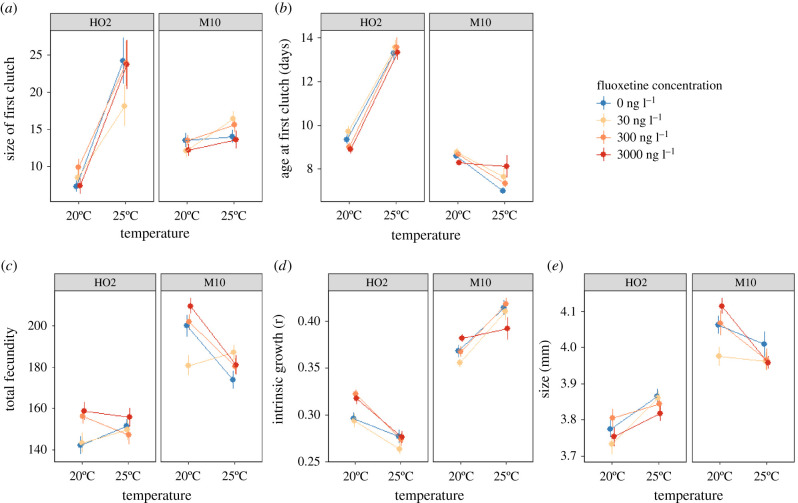
Univariate responses of two different genotypes (HO2 and M10) of *Daphnia magna* exposed to four different fluoxetine treatments (0 ng l^−1^, 30 ng l^−1^, 300 ng l^−1^ and 3000 ng l^−1^) at two different temperatures (20°C to 25°C). Means with standard error bars are depicted. (Online version in colour.)

We found that responses were often specific to the trait measured, concentration of fluoxetine, temperature and *Daphnia* genotype. The simplest effects were observed for the timing and size of first clutch, whereby fluoxetine had no significance effect on trait values, while the influence of temperature was much stronger for genotype HO2, accounting for the genotype by temperature interaction (table 1 and figure 1*a,b*). For all other traits, the influence of fluoxetine depended either on temperature (temperature × fluoxetine interaction, table 1) or the interplay between both temperature and genotype (three-way interaction, table 1). At lower temperatures, we typically observed far greater differences among the fluoxetine treatments than at higher temperatures. At 20°C, we saw that HO2 *Daphnia* exposed to the two higher fluoxetine concentrations had higher fecundity and intrinsic growth. For M10, we observed a non-monotonic response whereby individuals exposed to the lowest fluoxetine concentration had suppressed fecundity and body size relative to the controls, while the highest fluoxetine concentration greatly increased these traits. By contrast, at 25°C we found that for both genotypes there were no significant differences in fecundity, body size and intrinsic growth across the different fluoxetine treatments (figure 1*c,d,e*).

**Table 1.** Effects of genotype, temperature and fluoxetine treatment, as well as interactions between these terms, on total offspring, bodysize, age at first clutch, size of first clutch and intrinsic growth of *Daphnia magna.* Analysis was performed using linear mixed effect models on each trait.

trait	term	*χ*^2^	d.f.	*p*-value
*size of first clutch*	genotype	3.049	1	0.081
	temperature	88.942	1	*<0*.*001*
	fluoxetine treatment	2.772	3	0.428
	genotype : temperature	49.495	1	*<0*.*001*
	genotype : fluoxetine treatment	2.701	3	0.440
	temperature : fluoxetine treatment	0.749	3	0.862
	genotype : temperature : fluoxetine treatment	6.164	3	0.104
*age at first clutch*	genotype	382.985	1	*<0*.*001*
	temperature	129.106	1	*<0*.*001*
	fluoxetine treatment	5.174	3	0.160
	genotype : temperature	165.404	1	*<0*.*001*
	genotype : fluoxetine treatment	1.146	3	0.766
	temperature : fluoxetine treatment	3.130	3	0.372
	genotype : temperature : fluoxetine treatment	2.217	3	0.529
*total offspring*	genotype	315.303	1	*<0*.*001*
	temperature	14.128	1	*<0*.*001*
	fluoxetine treatment	15.317	3	*0*.*002*
	genotype : temperature	17.900	1	*<0*.*001*
	genotype : fluoxetine treatment	0.232	3	0.972
	temperature : fluoxetine treatment	17.062	3	*0*.*001*
	genotype : temperature : fluoxetine treatment	9.550	3	*0*.*023*
*intrinsic growth (r)*	genotype	807.281	1	*<0*.*001*
	temperature	0.735	1	0.391
	fluoxetine treatment	9.629	3	*0*.*022*
	genotype : temperature	118.680	1	*<0*.*001*
	genotype : fluoxetine treatment	3.137	3	0.371
	temperature : fluoxetine treatment	11.400	3	*0*.*010*
	genotype : temperature : fluoxetine treatment	6.822	3	0.078
*body size*	genotype	278.676	1	*<0*.*001*
	temperature	0.000	1	0.986
	fluoxetine treatment	7.835	3	0.050
	genotype : temperature	41.916	1	*<0*.*001*
	genotype : fluoxetine treatment	5.709	3	0.127
	temperature : fluoxetine treatment	10.987	3	*0*.*012*
	genotype : temperature : fluoxetine treatment	1.879	3	0.598

### (b) Multivariate analysis indicates an antagonistic interaction between temperature and fluoxetine exposure for both genotypes

Across the different life-history traits, we observed a variety of responses to genotype, fluoxetine and temperature. After accounting for correlations among trait responses via a phenotypic trajectory analysis, however, we observed an overarching antagonistic interaction between temperature and fluoxetine concentration. We found no significant difference in the magnitude of the phenotype trajectories between *Daphnia* exposed to different fluoxetine treatments at 20°C and 25°C (*D* in table 2), indicating that the strength of temperature-driven phenotypic change is not altered by fluoxetine exposure, at both ecologically relevant and extreme concentrations. Instead, there were significant differences in the angles of the phenotype trajectories for each fluoxetine treatment (*θ* in table 2), particularly for the M10 genotype of *Daphnia* whereby most angles were greater than 30°. This indicated a reduction of phenotype differences at 25°C compared to 20°C.

**Table 2.** Phenotypic trajectory analysis (PTA) showing differences in magnitude (*D*) and angle (*θ*) in temperature driven phenotype shifts across each fluoxetine treatment comparison within each genotype. Phenotypes are based on five life-history traits.

genotype	treatment comparisons	magnitude difference (*D*)	*Z*	*p*-value	angle difference (*θ*)	*Z*	*p*-value
HO2	0 ng l^−1^ : 30 ng l^−1^	0.079	−0.891	0.792	18.859	0.378	0.322
	0 ng l^−1^ : 300 ng l^−1^	0.280	0.128	0.387	31.642	2.128	*0*.*034*
	0 ng l^−1^ : 3000 ng l^−1^	0.227	−0.172	0.482	21.349	0.679	0.235
	30 ng l^−1^ : 300 ng l^−1^	0.359	0.508	0.263	30.102	1.909	*0*.*044*
	30 ng l^−1^ : 3000 ng l^−1^	0.307	0.202	0.372	22.757	0.885	0.188
	300 ng l^−1^ : 3000 ng l^−1^	0.053	−1.057	0.882	10.540	−0.844	0.787
M10	0 ng l^−1^ : 30 ng l^−1^	0.067	−1.019	0.851	41.647	2.659	*0*.*011*
	0 ng l^−1^ : 300 ng l^−1^	0.061	−1.107	0.880	15.653	−0.491	0.659
	0 ng l^−1^ : 3000 ng l^−1^	0.467	0.511	0.267	39.854	2.272	*0*.*025*
	30 ng l^−1^ : 300 ng l^−1^	0.129	−0.771	0.747	36.260	1.964	*0*.*038*
	30 ng l^−1^ : 3000 ng l^−1^	0.534	0.916	0.181	64.922	5.325	*0*.*001*
	300 ng l^−1^ : 3000 ng l^−1^	0.406	0.373	0.322	32.108	1.370	0.104

**Figure 2.**
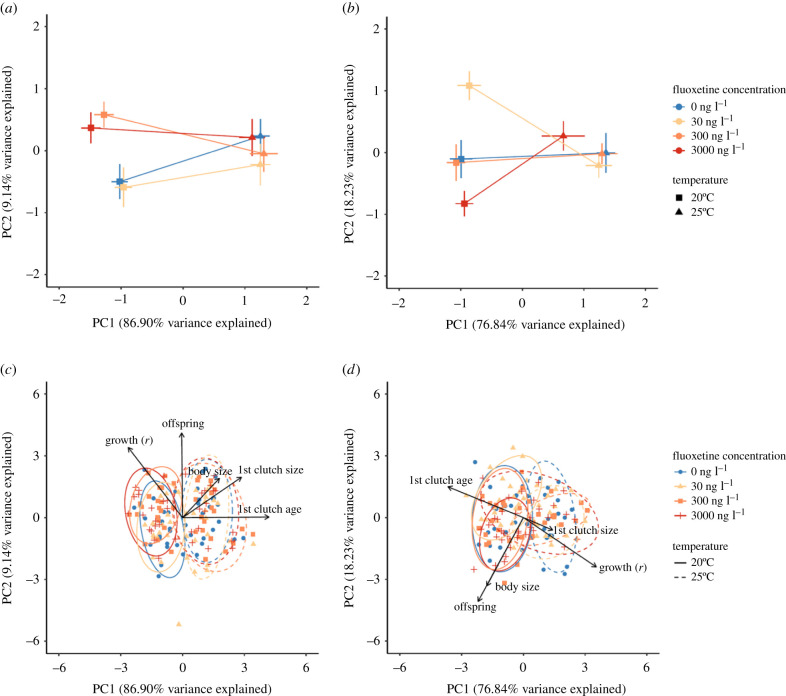
Principal component plots depicting: (*a*) phenotype trajectories of HO2 genotype *Daphnia magna* in response to temperature and fluoxetine treatments, (*b*) phenotype trajectories of M10 genotype *Daphnia*. (*c*) All observations for HO2 *Daphnia* grouped according to temperature and fluoxetine treatments and (*d*) all observations for M10 *Daphnia* grouped according to temperature and fluoxetine treatments. Contributions of each life-history trait (first clutch age, first clutch size, offspring, body size and intrinsic growth) toward the PC1 and PC2 axis for each genotype are shown (*c*,*d*). Ellipses represent 95% confidence bands. (Online version in colour.)

Visualization of the phenotypic trajectory analysis revealed that increases in temperature lead to a convergence of life-history phenotypes and a reduction of phenotype differences at 25°C compared to 20°C (figure 2). The phenotype trajectories of each fluoxetine treatment diverged along the PC2 axis at 20°C, which primarily accounts for variation in total offspring and intrinsic growth for HO2 (figure 2*c*), and total offspring and body size for M10 (figure 2*d*). These trajectories then converged at 25°C (figure 2*a,b*), with increased temperature described by large shifts across the PC1 axis (figure 2*a,b*), which, for HO2, is primarily driven by differences in size and timing of the first clutch (figure 2*c*), and, for M10, intrinsic growth as well as timing of the first clutch (figure 2*d*).

## 4. Discussion

We found that even trace amounts of fluoxetine can affect a variety of life-history traits in *Daphnia*. While previous studies have indicated that fluoxetine exposure can alter fecundity in *Daphnia* [47,57] as well as other invertebrates [66,67], effects at exposure concentrations lower than 10 µg have rarely been seen before. We observed that ecologically important traits such as fecundity, body size and intrinsic growth were all affected by the lowest concentration of fluoxetine (30 ng l^−1^). In specific cases (*Daphnia* genotype M10 for example), fluoxetine even had a non-monotonic effect on fecundity and body size, whereby the lowest concentration induced the greatest phenotypic change. Non-monotonic responses are increasingly being reported in studies investigating the effects of fluoxetine on wildlife (e.g. [68–71]), possibly because, at higher concentrations, receptors become desensitized, or negative feedback loops are induced [72]. Our results highlight the need to use environmentally realistic concentrations when investigating the effects of pharmaceuticals on ecosystems, as effects at these concentrations may be notably different and sometimes more severe than effects seen at the higher concentrations typical of many studies.

The introduction of another ecological challenge, thermal stress, fundamentally altered the phenotypic consequences of fluoxetine exposure. A 5°C increase in temperature led to all treatments, freshwater control and fluoxetine exposures alike, converging on a common life-history phenotype (figure 2), suggesting that rising temperatures may potentially reduce the net phenotypic effects of fluoxetine pollution on an individual's life-history in some contexts. One potential explanation for this process is that fluoxetine is eliminated more rapidly as temperature increases [73,74], reducing its impact. Increases in temperate are known to accelerate the pace of life for an organism, favouring higher reproductive turn over and shorter lifespans [75], and in this case perhaps the rapid elimination of fluoxetine. It is also possible that increased temperature may disrupt the mechanism via which fluoxetine induces a response, as has been seen in other examples of antagonistic interactions between temperature and chemical pollutants [41,76]. Regardless of the underlying mechanism, our results suggest that the effects of the pollutant fluoxetine will not necessarily be exacerbated under the rise in temperatures predicted for many scenarios of global change. This demonstrates that while climate change is often predicted to amplify threats to ecosystems, this is not always inevitable (see also [39,77]), although due to the complex nature of ecosystems, the exact effects are likely to depend on context, such as the type of pollutant, the type of thermal change, and as we discuss below, the genetic background of the exposed individual. In particular, in any lake or pond where *Daphnia* exist, they will likely be exposed to a variety of temperatures, due to spatial and temporal variation in thermal regimes, and their own ability to migrate vertically in the water column [78–80]. In the wild, the potential for temperature change to limit the impact of fluoxetine will depend strongly on this fine-scale variation in temperature and the exposed individual's own thermal preference.

While very few studies have investigated interactions between temperature and fluoxetine specifically, the studies that exist have yielded conflicting results. Barbosa *et al.* [81], for example, found that fluoxetine exposure and increased variation in temperature had a synergistic effect on *Daphnia* lifetime reproductive success and population growth rate, while Wiles *et al.* [82] found no interactions between temperature stress and fluoxetine exposure on guppy behaviour. Given that, in our study, fluoxetine by temperature responses appeared to be trait-specific under the univariate analyses, we suggest that these contrasting results should be expected on a trait-by-trait basis. As our multivariate analysis revealed, it is only by integrating across many traits that a consensus may emerge for how interactions between fluoxetine and other ecological relevant stressors might influence phenotypic change in a population. Otherwise, viewing the effect of pollutants on single traits in isolation may fundamentally under or overestimate the consequences of pharmaceutical pollutants for natural populations.

We also found that the effects of fluoxetine and temperature were often affected by the genotype of *Daphnia*, a factor that has rarely been considered when investigating effects of pharmaceutical pollutants. More commonly the effects of toxicants are typically tested using only a single standard background genotype ([83–87] but see [52]), overlooking a considerable source of variation underlying a population's response to pharmaceuticals. While we only examined two genotypes, these have been shown to vary considerably in a variety of contexts [49,50,58], and, therefore, give an indication of the potential for genotype-specific responses. Employing a variety of genotypes will help to further explore how genetic variability could shape a population's net response to pharmaceutical pollution, as well as its potential to evolve in response to this source of human-induced environmental change.

Overall, our findings highlight the complexity of wildlife responses to chemical pollutants, where secondary factors such as temperature can fundamentally alter phenotypic consequences in unforeseen ways. Indeed, warmer temperatures appear to lessen the effects of fluoxetine on an organism's life history, suggesting that the effects of this widespread pharmaceutical will not necessarily be made worse under common scenarios of global change. Such a result could easily have been overlooked if only a single host trait was measured, if host genotype had not been taken into account, or if ecologically relevant concentrations of the pollutant were not employed. Accordingly, to understand the full impact of pharmaceuticals on wildlife, we suggest that future studies capture more of the complexity of natural populations, where genetic variability, complex multivariate phenotypes, and the potential for non-monotonic responses, interact to shape individual performance or overall ecosystem function in the face of pharmaceutical pollutants.

## Data accessibility

Data are available from the Dryad Digital Repository: https://doi.org/10.5061/dryad.8w9ghx3mh [88].

## References

[1] Küster A, Adler N. 2014 Pharmaceuticals in the environment: scientific evidence of risks and its regulation. Phil. Trans. R. Soc. B **369**, 20130587. (10.1098/rstb.2013.0587) 25405974 PMC4213597

[2] Monteiro SC, Boxall AB. 2010 Occurrence and fate of human pharmaceuticals in the environment. Rev. Environ. Contam. Toxicol. **202**, 53-154. (10.1007/978-1-4419-1157-5_2) 19898761

[3] Kookana RS et al. 2014 Potential ecological footprints of active pharmaceutical ingredients: an examination of risk factors in low-, middle- and high-income countries. Phil. Trans. R. Soc. B **369**, 20130586. (10.1098/rstb.2013.0586) 25405973 PMC4213596

[4] Richmond EK, Rosi EJ, Walters DM, Fick J, Hamilton SK, Brodin T, Sundelin A, Grace MR. 2018 A diverse suite of pharmaceuticals contaminates stream and riparian food webs. Nat. Commun. **9**, 4491. (10.1038/s41467-018-06822-w) 30401828 PMC6219508

[5] Arnold KE, Brown AR, Ankley GT, Sumpter JP. 2014 Medicating the environment: assessing risks of pharmaceuticals to wildlife and ecosystems. Phil. Trans. R. Soc. B **369**, 20130569. (10.1098/rstb.2013.0569) 25405959 PMC4213582

[6] Fent K, Weston AA, Caminada D. 2006 Ecotoxicology of human pharmaceuticals. Aquat. Toxicol. **76**, 122-159. (10.1016/j.aquatox.2005.09.009) 16257063

[7] Saaristo M et al. 2018 Direct and indirect effects of chemical contaminants on the behaviour, ecology and evolution of wildlife. Proc. R. Soc. B **285**, 20181297. (10.1098/rspb.2018.1297) PMC6125903 30135169

[8] Johnson AC, Jin X, Nakada N, Sumpter JP. 2020 Learning from the past and considering the future of chemicals in the environment. Science **367**, 384-387. (10.1126/science.aay6637) 31974243

[9] Silva LJ, Lino CM, Meisel LM, Pena A. 2012 Selective serotonin re-uptake inhibitors (SSRIs) in the aquatic environment: an ecopharmacovigilance approach. Sci. Total Environ. **437**, 185-195. (10.1016/j.scitotenv.2012.08.021) 22940043

[10] Kolpin DW, Furlong ET, Meyer MT, Thurman EM, Zaugg SD, Barber LB, Buxton HT. 2002 Pharmaceuticals, hormones, and other organic wastewater contaminants in US streams, 1999−2000: a national reconnaissance. Environ. Sci. Technol. **36**, 1202-1211. (10.1021/es011055j) 11944670

[11] Benotti MJ, Brownawell BJ. 2007 Distributions of pharmaceuticals in an urban estuary during both dry-and wet-weather conditions. Environ. Sci. Technol. **41**, 5795-5802. (10.1021/es0629965) 17874789

[12] Frazer A. 2001 Serotonergic and noradrenergic reuptake inhibitors: prediction of clinical effects from *in vitro* potencies. J. Clin. Psychiatry **62**, 16. 11430614

[13] Stahl SM. 1998 Mechanism of action of serotonin selective reuptake inhibitors: serotonin receptors and pathways mediate therapeutic effects and side effects. J. Affect. Disord. **51**, 215-235. (10.1016/S0165-0327(98)00221-3) 10333979

[14] Ford AT, Fong PP. 2016 The effects of antidepressants appear to be rapid and at environmentally relevant concentrations. Environ. Toxicol. Chem. **35**, 794-798. (10.1002/etc.3087) 26031210

[15] Fong PP, Ford AT. 2014 The biological effects of antidepressants on the molluscs and crustaceans: a review. Aquat. Toxicol. **151**, 4-13. (10.1016/j.aquatox.2013.12.003) 24374179

[16] Conners DE, Rogers ED, Armbrust KL, Kwon JW, Black MC. 2009 Growth and development of tadpoles (*Xenopus laevis*) exposed to selective serotonin reuptake inhibitors, fluoxetine and sertraline, throughout metamorphosis. Environ. Toxicol. Chem. **28**, 2671-2676. (10.1897/08-493.1) 19572769

[17] Dzieweczynski TL, Hebert OL. 2012 Fluoxetine alters behavioral consistency of aggression and courtship in male Siamese fighting fish, *Betta splendens*. Physiol. Behav. **107**, 92-97. (10.1016/j.physbeh.2012.06.007) 22722098

[18] Martin JM, Saaristo M, Tan H, Bertram MG, Nagarajan-Radha V, Dowling DK, Wong BB. 2019 Field-realistic antidepressant exposure disrupts group foraging dynamics in mosquitofish. Biol. Lett. **15**, 20190615. (10.1098/rsbl.2019.0615) 31718515 PMC6892513

[19] Polverino G, Martin JM, Bertram MG, Soman VR, Tan H, Brand JA, Mason RT, Wong BB. 2021 Psychoactive pollution suppresses individual differences in fish behaviour. Proc. R. Soc. B **288**, 20202294. (10.1098/rspb.2020.2294) PMC7893217 33563120

[20] Aulsebrook LC et al. 2020 Reproduction in a polluted world: implications for wildlife. Reproduction **160**, R13-R23. (10.1530/REP-20-0154) 32442963

[21] Straub L, Strobl V, Neumann P. 2020 The need for an evolutionary approach to ecotoxicology. Nat. Ecol. Evol. **4**, 895. (10.1038/s41559-020-1194-6) 32327743

[22] Thoré ES, Van Hooreweghe F, Philippe C, Brendonck L, Pinceel T. 2021 Generation-specific and interactive effects of pesticide and antidepressant exposure in a fish model call for multi-stressor and multigenerational testing. Aquat. Toxicol. **232**, 105743. (10.1016/j.aquatox.2021.105743) 33460950

[23] Crain CM, Kroeker K, Halpern BS. 2008 Interactive and cumulative effects of multiple human stressors in marine systems. Ecol. Lett. **11**, 1304-1315. (10.1111/j.1461-0248.2008.01253.x) 19046359

[24] Liess M, Foit K, Knillmann S, Schäfer RB, Liess H-D. 2016 Predicting the synergy of multiple stress effects. Sci. Rep. **6**, 1-8. (10.1038/srep32965) 27609131 PMC5017025

[25] Folt C, Chen C, Moore M, Burnaford J. 1999 Synergism and antagonism among multiple stressors. Limnol. Oceanogr. **44**(3part2), 864-877. (10.4319/lo.1999.44.3_part_2.0864)

[26] Hall MD, Vettiger A, Ebert D. 2013 Interactions between environmental stressors: the influence of salinity on host–parasite interactions between *Daphnia magna* and *Pasteuria ramosa*. Oecologia **171**, 789-796. (10.1007/s00442-012-2452-3) 23001624

[27] Relyea RA, Mills N. 2001 Predator-induced stress makes the pesticide carbaryl more deadly to gray treefrog tadpoles (*Hyla versicolor*). Proc. Natl Acad. Sci. USA **98**, 2491-2496. (10.1073/pnas.031076198) 11226266 PMC30165

[28] Holmstrup M et al. 2010 Interactions between effects of environmental chemicals and natural stressors: a review. Sci. Total Environ. **408**, 3746-3762. (10.1016/j.scitotenv.2009.10.067) 19922980

[29] Henry M, Bertrand C, Le Féon V, Requier F, Odoux J-F, Aupinel P, Bretagnolle V, Decourtye A. 2014 Pesticide risk assessment in free-ranging bees is weather and landscape dependent. Nat. Commun. **5**, 1-8. (10.1038/ncomms5359) 25008773

[30] Rohr JR et al. 2008 Agrochemicals increase trematode infections in a declining amphibian species. Nature **455**, 1235-1239. (10.1038/nature07281) 18972018

[31] Blanck H. 2002 A critical review of procedures and approaches used for assessing pollution-induced community tolerance (PICT) in biotic communities. Hum. Ecol. Risk Assess. **8**, 1003-1034. (10.1080/1080-700291905792)

[32] Huey RB, Stevenson R. 1979 Integrating thermal physiology and ecology of ectotherms: a discussion of approaches. Am. Zool. **19**, 357-366. (10.1093/icb/19.1.357)

[33] Hansen J, Sato M, Ruedy R, Lo K, Lea DW, Medina-Elizade M. 2006 Global temperature change. Proc. Natl Acad. Sci. USA **103**, 14 288-14 293. (10.1073/pnas.0606291103) PMC1576294 17001018

[34] Gordon CJ. 2003 Role of environmental stress in the physiological response to chemical toxicants. Environ. Res. **92**, 1-7. (10.1016/S0013-9351(02)00008-7) 12706749

[35] Besson M et al. 2020 Anthropogenic stressors impact fish sensory development and survival via thyroid disruption. Nat. Commun. **11**, 1-10. (10.1038/s41467-020-17450-8) 32681015 PMC7367887

[36] Laetz CA, Baldwin DH, Hebert VR, Stark JD, Scholz NL. 2014 Elevated temperatures increase the toxicity of pesticide mixtures to juvenile coho salmon. Aquat. Toxicol. **146**, 38-44. (10.1016/j.aquatox.2013.10.022) 24270668

[37] Cardoso P, Rodrigues D, Madureira T, Oliveira N, Rocha M, Rocha E. 2017 Warming modulates the effects of the endocrine disruptor progestin levonorgestrel on the zebrafish fitness, ovary maturation kinetics and reproduction success. Environ. Pollut **229**, 300-311. (10.1016/j.envpol.2017.05.090) 28601762

[38] Perschbacher PW, Wurts WA. 1999 Effects of calcium and magnesium hardness on acute copper toxicity to juvenile channel catfish, *Ictalurus punctatus*. Aquaculture **172**, 275-280. (10.1016/S0044-8486(98)00499-2)

[39] Zhang C, Jansen M, De Meester L, Stoks R. 2019 Rapid evolution in response to warming does not affect the toxicity of a pollutant: insights from experimental evolution in heated mesocosms. Evol. Appl. **12**, 977-988. (10.1111/eva.12772) 31080509 PMC6503828

[40] Abdel-Lateif H, Donker M, Van Straalen N. 1998 Interaction between temperature and cadmium toxicity in the isopod *Porcellio scaber*. Funct. Ecol. **12**, 521-527. (10.1046/j.1365-2435.1998.00227.x)

[41] Talent LG. 2005 Effect of temperature on toxicity of a natural pyrethrin pesticide to green anole lizards (*Anolis carolinensis*). Environ. Toxicol. Chem. **24**, 3113-3116. (10.1897/05-053R.1) 16445093

[42] Stoks R, Govaert L, Pauwels K, Jansen B, De Meester L. 2016 Resurrecting complexity: the interplay of plasticity and rapid evolution in the multiple trait response to strong changes in predation pressure in the water flea *Daphnia magna*. Ecol. Lett. **19**, 180-190. (10.1111/ele.12551) 26647739

[43] Plaistow S, Collin H. 2014 Phenotypic integration plasticity in *Daphnia magna*: an integral facet of G × E interactions. J. Evol. Biol. **27**, 1913-1920. (10.1111/jeb.12443) 25099216

[44] Collyer ML, Adams DC. 2007 Analysis of two-state multivariate phenotypic change in ecological studies. Ecology **88**, 683-692. (10.1890/06-0727) 17503596

[45] Collyer ML, Sekora DJ, Adams DC. 2015 A method for analysis of phenotypic change for phenotypes described by high-dimensional data. Heredity **115**, 357-365. (10.1038/hdy.2014.75) 25204302 PMC4815463

[46] Adams DC, Collyer ML. 2009 A general framework for the analysis of phenotypic trajectories in evolutionary studies. Evolution **63**, 1143-1154. (10.1111/j.1558-5646.2009.00649.x) 19210539

[47] Flaherty CM, Dodson SI. 2005 Effects of pharmaceuticals on *Daphnia* survival, growth, and reproduction. Chemosphere **61**, 200-207. (10.1016/j.chemosphere.2005.02.016) 16168743

[48] Ebert D, 2005 Ecology, epidemiology, and evolution of parasitism in Daphnia. Bethesda, MD: National Library of Medicine (US), National Center for Biotechnololgy Information.

[49] Hall MD, Ebert D. 2012 Disentangling the influence of parasite genotype, host genotype and maternal environment on different stages of bacterial infection in *Daphnia magna*. Proc. R. Soc. B **279**, 3176-3183. (10.1098/rspb.2012.0509) PMC3385728 22593109

[50] Michel J, Ebert D, Hall MD. 2016 The trans-generational impact of population density signals on host–parasite interactions. BMC Evol. Biol. **16**, 1-12. (10.1186/s12862-016-0828-4) 27887563 PMC5123254

[51] Brans KI, De Meester L. 2018 City life on fast lanes: urbanization induces an evolutionary shift towards a faster lifestyle in the water flea *Daphnia*. Funct. Ecol. **32**, 2225-2240. (10.1111/1365-2435.13184)

[52] Sadler DE, Brunner FS, Plaistow SJ. 2019 Temperature and clone-dependent effects of microplastics on immunity and life history in *Daphnia magna*. Environ. Pollut **255**, 113178. (10.1016/j.envpol.2019.113178) 31520904

[53] Hector TE, Sgrò CM, Hall MD. 2021 Temperature and pathogen exposure act independently to drive host phenotypic trajectories. Biol. Lett. **17**, 20210072. (10.1098/rsbl.2021.0072) 34129797 PMC8205525

[54] Mole RA, Brooks BW. 2019 Global scanning of selective serotonin reuptake inhibitors: occurrence, wastewater treatment and hazards in aquatic systems. Environ. Pollut **250**, 1019-1031. (10.1016/j.envpol.2019.04.118) 31085468

[55] Péry A, Gust M, Vollat B, Mons R, Ramil M, Fink G, Ternes T, Garric J. 2008 Fluoxetine effects assessment on the life cycle of aquatic invertebrates. Chemosphere **73**, 300-304. (10.1016/j.chemosphere.2008.06.029) 18656226

[56] Hansen LK, Frost PC, Larson JH, Metcalfe CD. 2008 Poor elemental food quality reduces the toxicity of fluoxetine on *Daphnia magna*. Aquat. Toxicol. **86**, 99-103. (10.1016/j.aquatox.2007.10.005) 18037510

[57] Campos B, Pina B, Fernández-Sanjuán M, Lacorte S, Barata C. 2012 Enhanced offspring production in *Daphnia magna* clones exposed to serotonin reuptake inhibitors and 4-nonylphenol. Stage- and food-dependent effects. Aquat. Toxicol. **109**, 100-110. (10.1016/j.aquatox.2011.12.003) 22210498

[58] Hector TE, Sgrò CM, Hall MD. 2019 Pathogen exposure disrupts an organism's ability to cope with thermal stress. Glob. Change Biol. **25**, 3893-3905. (10.1111/gcb.14713) 31148326

[59] Klüttgen B, Dülmer U, Engels M, Ratte H. 1994 ADaM, an artificial freshwater for the culture of zooplankton. Water Res. **28**, 743-746. (10.1016/0043-1354(94)90157-0)

[60] Ebert D, Zschokke-Rohringer CD, Carius HJ. 1998 Within- and between-population variation for resistance of *Daphnia magna* to the bacterial endoparasite *Pasteuria ramosa*. Proc. R. Soc. B **265**, 2127-2134. (10.1098/rspb.1998.0549)

[61] Fursdon JB, Martin JM, Bertram MG, Lehtonen TK, Wong BB. 2018 The pharmaceutical pollutant fluoxetine alters reproductive behaviour in a fish independent of predation risk. Sci. Total Environ. **650**, 642-652. (10.1016/j.scitotenv.2018.09.046) 30212693

[62] Bertram MG, Ecker TE, Wong BBM, O'Bryan MK, Baumgartner JB, Martin JM, Saaristo M. 2018 The antidepressant fluoxetine alters mechanisms of pre- and post-copulatory sexual selection in the eastern mosquitofish (*Gambusia holbrooki*). Environ. Pollut **238**, 238-247. (10.1016/j.envpol.2018.03.006) 29567445

[63] Shocket MS, Strauss AT, Hite JL, Šljivar M, Civitello DJ, Duffy MA, Cáceres CE, Hall SR. 2018 Temperature drives epidemics in a zooplankton–fungus disease system: a trait-driven approach points to transmission via host foraging. Am. Nat. **191**, 435-451. (10.1086/696096) 29570399

[64] R Core Team. 2020 R: a language and environment for statistical computing. Vienna, Austria: R Foundation for Statistical Computing. See http://www.R-project.org/.

[65] Collyer ML, Adams DC. 2018 RRPP: an r package for fitting linear models to high-dimensional data using residual randomization. Methods Ecol. Evol. **9**, 1772-1779. (10.1111/2041-210X.13029)

[66] Fong PP. 1998 Zebra mussel spawning is induced in low concentrations of putative serotonin reuptake inhibitors. Biol. Bull. **194**, 143-149. (10.2307/1543044) 28570848

[67] Brooks BW, Turner PK, Stanley JK, Weston JJ, Glidewell EA, Foran CM, Slattery M, La Point TW, Huggett DB. 2003 Waterborne and sediment toxicity of fluoxetine to select organisms. Chemosphere **52**, 135-142. (10.1016/S0045-6535(03)00103-6) 12729696

[68] Guler Y, Ford AT. 2010 Anti-depressants make amphipods see the light. Aquat. Toxicol. **99**, 397-404. (10.1016/j.aquatox.2010.05.019) 20591511

[69] De Lange H, Noordoven W, Murk A, Lürling M, Peeters E. 2006 Behavioural responses of *Gammarus pulex* (Crustacea, Amphipoda) to low concentrations of pharmaceuticals. Aquat. Toxicol. **78**, 209-216. (10.1016/j.aquatox.2006.03.002) 16624423

[70] Martin JM, Saaristo M, Bertram MG, Lewis PJ, Coggan TL, Clarke BO, Wong BBM. 2017 The psychoactive pollutant fluoxetine compromises antipredator behaviour in fish. Environ. Pollut **222**, 592-599. (10.1016/j.envpol.2016.10.010) 28063712

[71] Barry MJ. 2013 Effects of fluoxetine on the swimming and behavioural responses of the Arabian killifish. Ecotoxicology **22**, 425-432. (10.1007/s10646-012-1036-7) 23264030

[72] Vandenberg LN et al. 2012 Hormones and endocrine-disrupting chemicals: low-dose effects and nonmonotonic dose responses. Endocr Rev. **33**, 378-455. (10.1210/er.2011-1050) 22419778 PMC3365860

[73] Hooper MJ, Ankley GT, Cristol DA, Maryoung LA, Noyes PD, Pinkerton KE. 2013 Interactions between chemical and climate stressors: a role for mechanistic toxicology in assessing climate change risks. Environ. Toxicol. Chem. **32**, 32-48. (10.1002/etc.2043) 23136056 PMC3601417

[74] Gordon CJ, Johnstone AF, Aydin C. 2011 Thermal stress and toxicity. Compr. Physiol. **4**, 995-1016. (10.1002/cphy.c130046) 24944028

[75] Zwaan B, Bijlsma R, Hoekstra R. 1992 On the developmental theory of ageing. II. The effect of developmental temperature on longevity in relation to adult body size in *D.* *melanogaster*. Heredity **68**, 123-130. (10.1038/hdy.1992.19) 1548140

[76] Mehdi H, Bragg LM, Servos MR, Craig PM. 2019 Multiple stressors in the environment: the effects of exposure to an antidepressant (venlafaxine) and increased temperature on zebrafish metabolism. Front. Physiol. **10**, 1431. (10.3389/fphys.2019.01431) 31803073 PMC6877669

[77] Clark TD, Raby GD, Roche DG, Binning SA, Speers-Roesch B, Jutfelt F, Sundin J. 2020 Ocean acidification does not impair the behaviour of coral reef fishes. Nature **577**, 370-375. (10.1038/s41586-019-1903-y) 31915382

[78] Jacobs AF, Heusinkveld BG, Kraai A, Paaijmans KP. 2008 Diurnal temperature fluctuations in an artificial small shallow water body. Int. J. Biometeorol. **52**, 271-280. (10.1007/s00484-007-0121-8) 17926069 PMC2668566

[79] Paaijmans KP, Jacobs A, Takken W, Heusinkveld B, Githeko A, Dicke M, Holtslag A. 2008 Observations and model estimates of diurnal water temperature dynamics in mosquito breeding sites in western Kenya. Hydrol. Process.: Int. J. **22**, 4789-4801. (10.1002/hyp.7099)

[80] Hays GC. 2003 A review of the adaptive significance and ecosystem consequences of zooplankton diel vertical migrations. Hydrobiologia **503,** 163-170. (10.1007/978-94-017-2276-6_18)

[81] Barbosa M, Inocentes N, Soares AM, Oliveira M. 2017 Synergy effects of fluoxetine and variability in temperature lead to proportionally greater fitness costs in *Daphnia*: a multigenerational test. Aquat. Toxicol. **193**, 268-275. (10.1016/j.aquatox.2017.10.017) 29125953

[82] Wiles SC, Bertram MG, Martin JM, Tan H, Lehtonen TK, Wong BB. 2020 Long-term pharmaceutical contamination and temperature stress disrupt fish behavior. Environ. Sci. Technol. **54**, 8072-8082. (10.1021/acs.est.0c01625) 32551542

[83] de Oliveira LLD, Antunes SC, Gonçalves F, Rocha O, Nunes B. 2016 Acute and chronic ecotoxicological effects of four pharmaceuticals drugs on cladoceran *Daphnia magna*. Drug Chem. Toxicol. **39**, 13-21. (10.3109/01480545.2015.1029048) 25864724

[84] Barata C, Baird DJ. 2000 Determining the ecotoxicological mode of action of chemicals from measurements made on individuals: results from instar-based tests with *Daphnia magna* Straus. Aquat. Toxicol. **48**, 195-209. (10.1016/S0166-445X(99)00038-7) 10686326

[85] Bownik A, Sokołowska N, Ślaska B. 2018 Effects of apomorphine, a dopamine agonist, on *Daphnia magna*: imaging of swimming track density as a novel tool in the assessment of swimming activity. Sci. Total Environ. **635**, 249-258. (10.1016/j.scitotenv.2018.04.157) 29669297

[86] Dietrich S, Ploessl F, Bracher F, Laforsch C. 2010 Single and combined toxicity of pharmaceuticals at environmentally relevant concentrations in *Daphnia magna*—a multigenerational study. Chemosphere **79**, 60-66. (10.1016/j.chemosphere.2009.12.069) 20116828

[87] Hassold E, Backhaus T. 2009 Chronic toxicity of five structurally diverse demethylase-inhibiting fungicides to the crustacean *Daphnia magna*: a comparative assessment. Environ. Toxicol. Chem. **28**, 1218-1226. (10.1897/08-339.1) 19132812

[88] Aulsebrook LC, Wong BBM, Hall MD. 2022 Data from: Warmer temperatures limit the effects of antidepressant pollution on life history traits. Dryad Digital Repository. (10.5061/dryad.8w9ghx3mh) PMC8825998 35135347

